# Neuro-otology symptoms as the early sign in pediatric patient with a pineal gland tumor: a case report

**DOI:** 10.1016/j.radcr.2022.06.001

**Published:** 2022-06-23

**Authors:** Aan Dwi Prasetio, Putri Irsalina, Wardah Rahmatul Islamiyah, Djohan Ardiansyah

**Affiliations:** Department of Neurology, Faculty of Medicine Airlangga University, Dr. Soetomo General Hospital, Surabaya, East Java 60286, Indonesia

**Keywords:** Dizziness, Pineal gland tumors, Central origin vertigo, Children

## Abstract

Patients with pineal tumors are often asymptomatic and the symptoms depend on the location of the mass. In fact, around 3%-8% of pediatric brain tumor cases are pineal tumors. Children with pineal tumors may present with dizziness and vertigo as early signs. These symptoms are common conditions among 5-15 years old children and could probably lead to misdiagnoses. We present a case of 14-year-old who came to the emergency room of DR. Soetomo Hospital Surabaya with neurotologic symptoms. After a series of radiographic and laboratory examinations, he was diagnosed with germinoma. A ventriculoperitoneal shunt was performed in the emergency room and intravenous dexamethasone was administered.

## Introduction

The prevalence of dizziness and vertigo in children aged 5-15 years old over a 1-year period, at least one episode of vertigo or dizziness, is 18% [1]. Tumor of the pineal region accounts for 3%-8% of brain tumors in children. There is a wide variety of pineal masses, with the majority being germ cell and pineal parenchymal tumors. Symptoms can be asymptomatic and vary depending on the local tumor invasion, tumor progression, and compression of adjacent structures [2]. Although pineal tumors are mostly asymptomatic, several reports in the medical literature associate pineal tumors with headaches [3].

Symptoms of vertigo and dizziness in children with pineal tumors can cause delayed postural control and impaired coordination. However, diagnosis can be burdensome since pediatric patients often describe their symptoms inaccurately. They may find it difficult to characterize the onset of the attack and what provoked the complaint [4]. Moreover, patients diagnosed with this condition mostly do not present with a chief complaint of balance disorders [5], although the association between balance disorders with sensorineural hearing loss, syncope, and headache in children is significant [6].

## Case report

A 14-year-old boy came to the emergency room (ER) of DR. Soetomo Hospital Surabaya had difficulty standing up and felt the environment around him shaking for 1 month, accompanied by nausea and vomiting. The patient felt that the headache was getting worse, his vision was doubled, and he often looked sleepy during the day for 2 months. The patient falls asleep more easily during the day than at night. There were no history of tumor in this patient and also his family.

Neurological examination of GCS was E4V5M6, and pupils were isochoric, 3 mm/3 mm round, with positive light reflex bilaterally. Examination of eye movements revealed esotropic eye position, bilateral oculomotor palsy, bilateral abducens palsy, bilateral positive corneal reflex, upward gaze palsy, positive skewed test in the right eye, and papilledema in both eyes. Motor examination showed no lateralization. Cerebellar function examination was within normal limits. Epworth Sleepiness Scale (ESS) of the patient is 13.

**Fig. 1:**
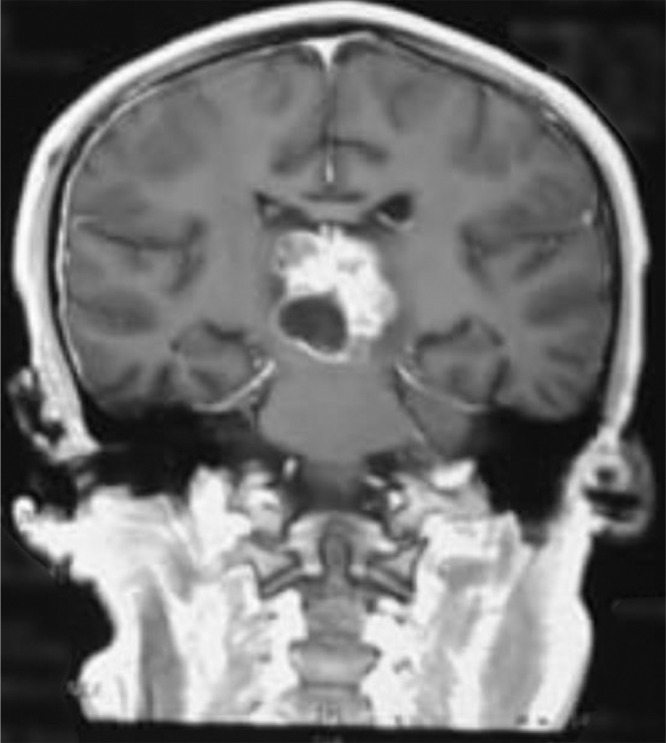
MRI T1 of the head with contrast (coronal section).

**Fig. 2:**
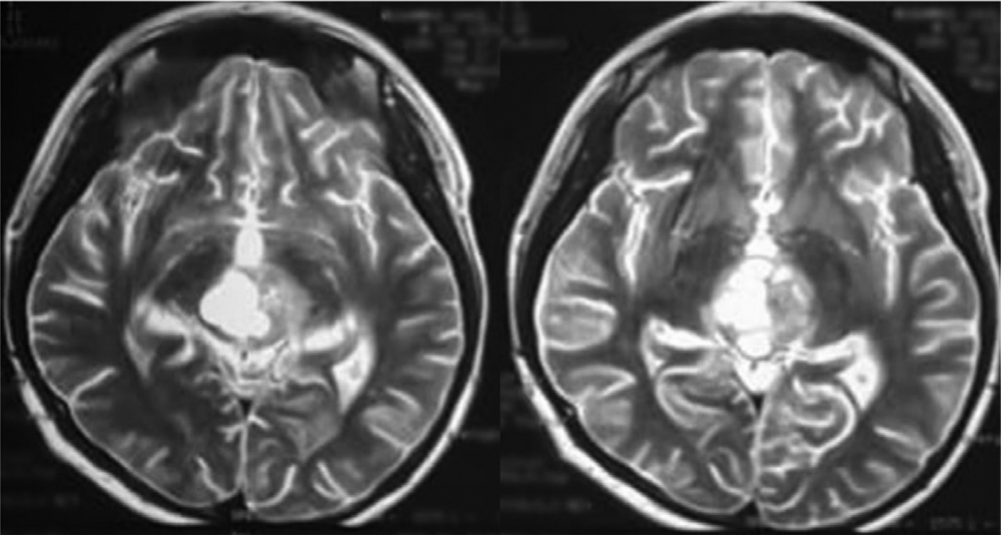
MRI T2 FLAIR of the head (axial section).

**Fig. 3:**
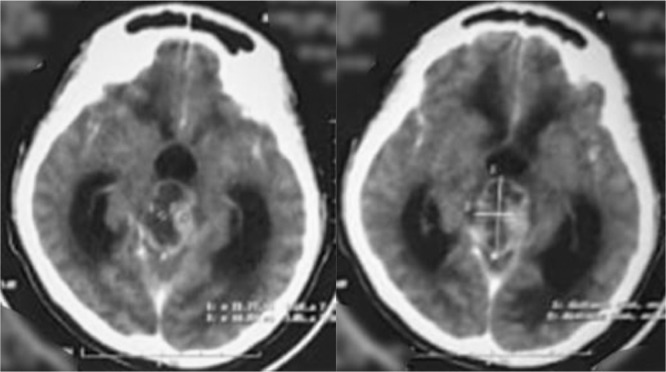
CT Scan of head with contrast (axial section).

Head computed tomography scan showed a supratentorial lesion with a cystic component of the pineal region which in contrast showed a solid portion with non-communicating hydrocephalus (Fig 1). Laboratory examination showed alpha-fetoprotein of <1.2 ng/mL and beta chorionic gonadotropin of 0.8 ng/mL, both were within the normal range. Lumbar puncture and cerebrospinal fluid cytology did not reveal any malignant cells. Head Magnetic resonance imaging (MRI) showed an intraventricular mass and dilatation of the third ventricle, supporting the pinealoma with a differential diagnosis of germinoma (Figs. 2 and 3). The patient had undergone a VP shunt as an emergency procedure to reduce intracranial pressure and received dexamethasone injection therapy. The results of anatomical pathology showed pineal germinoma, in which cells were arranged in sheets/nests/lobules separated by fibrovascular septa with lymphocyte cell filtration. Consists of a proliferation of large cells, pleomorphic, with a round nucleus, vesicular chromatin, prominent nucleoli, broad center, clear-pale eosinophilic. The patient was planned for therapy with chemotherapy and radiotherapy. Subjectively, the dizziness disorder in the patient improved, but the patient lost follow-up and refused further chemotherapy and radiotherapy on the grounds that his symptoms had improved.

## Discussion

It is essential to use advanced imaging techniques, together with clinical and laboratory knowledge to help differentiate between pineal neoplasms so as to assist in establishing an accurate diagnosis and proper treatment planning and patient management. Open and stereotactic surgical resection or endoscopic biopsy is required for the diagnosis of pineal tissue. However, stereotactic biopsy has the risk of bleeding is higher in tumors of the pineal region. In practice, the diagnosis of neoplasms in the pineal region is based on clinical presentation, imaging, and pathological results. Serum biomarkers and cerebrospinal fluid analysis can complement diagnostic data before invasive procedures are performed. Therefore, research on new diagnostic markers, therapeutic approaches, and follow-up guidelines is very important regarding tumor cases in the pineal region [7,8].

Dizziness and vertigo were common symptoms in 72% of children and one episode of vertigo typically occurs every 3 months. Compared to dizziness, a vertigo episode is often severe clinically because of its duration and impact on social activities. Meanwhile, both episodes of dizziness and vertigo may simultaneously occur in 30% of cases [4]. Functional dizziness, sensory disturbances–bilateral peripheral vestibular loss, and central vertigo can be recognized as persistent complaints and have specific clinical characteristics [9]. Central vertigo rarely occurs in children, but this diagnosis should not be disregarded since it is related to the prognosis of underlying diseases such as tumors [10]. Tumors of the pineal region of children can affect sleep patterns. It is associated with abnormal melatonin production. However, studies on pineal size, pineal disorders, and sleep disorders are scarce [3]. Vertigo, dizziness, and sleep disturbances are found as earlier signs in patients with a pineal tumor which is an important component in providing a differential diagnosis of a pineal tumor. Evaluation of sleep disorders using the ESS obtained a value of 13, meaning the patient is classified as having excessive sleepiness during the day which can be a sign of the presence of sleep disorders. To determine the type of sleep disorder, further examination would be carried out by a sleep specialist.

Germinomas are the most common pineal tumors, representing up to 50% of pineal tumors in Europe, the United States, and Japan. In a series of 370 pineal tumors in patients aged 3-73 years, it was observed that 27% were germinoma; 26% were astrocytomas; 12% were pineoblastomas; 12% were pineocytomas; 4.3% were ependymomas; 4.3% were teratomas; 2.7% were ganglioneuroma, lymphomas, meningiomas, metastases, and pineal cysts; 1.6% were mixed embryonal cell tumors (embryonic carcinoma)/malignant teratoma; 1.1% were choriocarcinomas; and 0.54% were oligodendrogliomas. Tumors in the pineal also cause non-specific signs and symptoms, 98% headaches, and signs of increased intracranial pressure, 57% have diabetes insipidus and growth retardation due to mass-pressure effects on the pituitary, 28% changes in vision, 15% impaired balance and coordination, and 10% memory impairment [11, 12].

The limitation of communication skills and vocabulary to explain symptoms become obstacles during the anamnesis. Meanwhile, the duration of complaints of vertigo and dizziness, as well as other symptoms such as nystagmus, hearing loss, and loss of consciousness should be explored further. Additionally, the history of illness during neonatal and perinatal periods related to the use of potentially ototoxic drugs should also be asked of the parents [13]. Since ataxia and strabismus are considered important findings in a child with nystagmus, neuroimaging was urgently performed to exclude a central lesion. The most common accompanying symptoms of nystagmus are headache and vertigo [5]. Motor deficits are merely the topographic diagnosis in central vestibular syndrome. Head MRI was performed if the clinical examination showed signs of ocular motor disturbances [4]. The patient has esotropic eyeball position, bilateral oculomotor palsy, bilateral abducens palsy, bilateral positive corneal reflex, upward gaze palsy, positive skewed test in the right eye, and papilledema in both eyes.

Tumors of the pineal region have a distinguished clinical onset and are associated with headache and vertigo, a clinical sign of intracranial hypertension. These symptoms are warning signs that require radiological examination. Non-communicating hydrocephalus due to compression of the cerebral aqueduct is another common clinical presentation in patients with tumors of the pineal region. Emergency measures, such as a ventriculostomy or ventriculoperitoneal shunt, are required in almost half of the cases [14].

According to Zhang et al., spontaneous regression of germinoma is a rare phenomenon. Only 10 cases were reported consisting of one female and 9 males, with a mean age of 22.1 ± 10.3 years (range: 12-43 years). Among these cases, 9 were diagnosed as germinoma, and 4 cases showed regression followed by regrowth. Zhang et al proposed 4 hypotheses to explain this regression, including radiation exposure, surgical procedures, effects of steroids, and immunotherapy. No clinical details are available in this patient; however, clinical improvement in our patient occurred after the patient underwent a VP shunt and steroid administration [7].

The gold standard diagnosis for pineal tumors is best with brain MRI, which has high sensitivity. MRI is the most accurate method for identifying tumors and describing their relationship to adjacent structures, allowing the true pineal mass to be distinguished from the parapineal mass. Awa et al described a study of 93 patients to clarify imaging features for differentiation of pineal germinoma and other pineal region tumors classified as germinoma, non-germinomatous germ cell tumors, pineal parenchymal tumors, and other tumors of the pineal region. Tumor extension in both thalamic and peritumoral edema is specific for differentiating germinoma from non-germinomatous germ cell tumors and pineal parenchymal tumors [7,11,15].

Mass in the anterior-inferior pineal region adjacent to the mesencephalon and posteriorly adjacent to the third ventricle. Tumor size at initial diagnosis has prognostic implications [14]. Tumors with less than 10 mm in size are considered asymptomatic, whereas tumors larger than 10 mm in size are considered symptomatic, therefore providing risk for neurological problems [3]. Head MRI is the gold standard for assessing pineal masses. It is useful for distinguishing between the pineal and parapineal masses from an invasion of the gland [2]. Head MRI showed a mass in the pineal region pressing on the surrounding structures including the central vestibular pathway, with explained the patient's complaints of central vertigo. Head MRI with contrast showed an intraventricular mass causing dilatation of the third ventricle. We immediately consulted the patient with the neurosurgery department, and a VP shunt was decided to be performed. Then, the symptoms improved. Asymptomatic treatment is useful for improving ataxia and postural imbalance [16]. The patient underwent a ventriculoperitoneal shunt to reduce intracranial pressure. The patient refused to continue hospitalization and further management of chemotherapy and radiotherapy. As a result, there is no follow-up data on the patient's current condition.

The interesting thing, in this case, is that the symptoms of balance disorders in children that are rarely realized by most parents can be a sign of a tumor that if not immediately followed up will endanger lives because of its progression. In addition, clinicians are expected to be more aware of each symptom, be careful and consider appropriate imaging when finding a child with vertigo symptoms. The shortcomings of this patient discussion relate to follow-up and clinical improvement and the risk of relapse in children with germinoma. However, after the patient had experienced an improvement in symptoms of balance disorders. We suspect this could be due to the post-VP shunt-lowering effect of intracranial pressure and the effects of steroids.

## Conclusions

In conclusion, vertigo and dizziness are common problems in children. Although the etiology is variable and most often benign, a central cause must also be considered. By combining complete anamnesis data, neurotology examinations, laboratory examinations, and radiological examinations to predict the direction of diagnosis, misdiagnosis can be minimalized.

## Data availability

All data underlying the results are available as part of the article and no additional source data are required.

## Patient consent statement

Written informed consent for publication of clinical details and clinical images was obtained from the patient.

## Author contributions

Putri Irsalina: Conceptualization, Investigation, Methodology, Writing – Original Draft Preparation; Aan Dwi Prasetio: Conceptualization, Investigation, Methodology, Writing – Original Draft Preparation; Wardah Rahmatul Islamiyah: Conceptualization, Investigation, Methodology, Supervision, Writing – Review & Editing; Djohan Ardiansyah: Conceptualization, Investigation, Methodology, Writing – Review & Editing.
